# Knowledge sharing during public health emergencies: from global call to effective implementation

**DOI:** 10.2471/BLT.16.172650

**Published:** 2016-04-01

**Authors:** Sophie Delaunay, Patricia Kahn, Mercedes Tatay, Joanne Liu

**Affiliations:** a Médecins Sans Frontières, 333 Seventh Ave, New York, NY 10001, United States of America.; b Médecins Sans Frontières, Geneva, Switzerland.

In February 2016, the issue of data sharing during emergencies made headlines around the world after leading research funders, academic journals and nongovernmental organizations signed a joint declaration of commitment to rapidly share data relevant to the Zika virus outbreak.^1^ This action followed repeated calls from some of the same constituencies for sharing data from clinical trials^2^^,^^3^ conducted in the context of public health emergencies^4^^,^^5^ and public health in general.^6^^,^^7^

While the Zika open data initiative is a positive step, it also highlights the shortcomings of calling for knowledge sharing after an outbreak has already begun. To improve epidemic emergency response and to accelerate related research, health authorities in potentially exposed countries must put in place the necessary frameworks for collecting, managing and swiftly making available good-quality, standardized data and for safely securing and sharing biomaterial­ – such as patient samples – collected during the outbreak.

The 2015 Ebola virus disease outbreak that took the lives of over 11 000 people offers ample lessons on why more knowledge sharing is essential and on how to achieve it. During the Ebola outbreak, massive amounts of data and biomaterials were collected. These included clinical and laboratory data from over 20 000 patients­­, some enrolled in clinical trials, tens of thousands of patient specimens and over two years’ worth of epidemiological data. If appropriate knowledge-sharing frameworks had been in place, these collections could have offered an historic opportunity to expand what is known about Ebola. One framework would guide data standardization, sharing, access and use, including rules on the transfer of agreed data sets into common repositories for curation. Another framework would facilitate cataloguing of biomaterials collected during an outbreak and would establish transparent rules for their management, thereby creating a virtual biobank. For future emergencies, a biobank framework will also need to encompass ethics guidelines for the collection and use of samples, including the provision of appropriate information to patients. 

Currently there are no mechanisms in place to advance such knowledge sharing. As the Zika outbreak shows, the global public health community is still unprepared to collect good quality, standardized data and biomaterials during emergencies and to share them in ways that provide equitable access to researchers. This limits researchers’ ability to optimize the use of available data and specimens in ways that fill key knowledge gaps and to ensure that research will benefit the affected patients and communities where the materials originated.

To address these shortcomings, Médecins Sans Frontières (MSF), in collaboration with the World Health Organization (WHO), has called upon stakeholders to establish a coordinated network of Ebola biobanks. Additionally, MSF has joined Oxford University’s Infectious Diseases Data Observatory to establish a data-sharing platform for existing and future clinical, biological and epidemiological data, with the aim of making this information accessible to stakeholders and researchers with relevant scientific questions. Together, a virtual biobank and a data repository could provide a global resource for the essential research needed to plan effective outbreak responses.

Neither the data sharing nor the biobank proposal is radically new. Data sharing is a long-accepted practice in many health research fields, from the Global Burden of Disease collaboration^8^ to surveillance and response to influenza, drug-resistant malaria and severe acute respiratory syndrome (SARS). Biobanks are well-established resources for disease research, for example on human immunodeficiency virus (HIV)^9^ and rare diseases.^10^ These arrangements benefit patients, researchers and the private sector. 

Our proposal for data and biomaterial frameworks raises many challenges that must be addressed,^5^^,^^6^^,^^11^^,^^12^ including technical and ethical concerns and fears that some benefits of sharing data or patient samples will go primarily to wealthy countries. However, these challenges only highlight the need for agreeing prospectively on transparent, ethical principles to guide the collection and future use of data and biomaterials collected for emergency health care. 

Drawing on existing models, such as the NIH platform,^13^ advocates for knowledge sharing in emergencies should emphasize the need to build the relevant frameworks before or at the onset of an outbreak. Since the most significant research gaps exist for diseases found in low- and middle-income countries, these frameworks should address each existing and emerging pathogen, rather than only those that threaten high-income countries. Building on its own call for action,^5^ WHO is the appropriate leader for such efforts.

In May 2016, when health ministers gather at the World Health Assembly, outbreak response will be high on the agenda. As they discuss WHO’s forward-thinking research and development blueprint for epidemics^14^ and consider strategies for improving emergency preparedness and response, delegates should also make a bold commitment to develop strong, equitable mechanisms that translate calls for sharing data and biomaterials into critically-needed action.
